# Deciphering the molecular network of Trichostatin A in regulating Alzheimer’s disease screening of core genes and mechanistic investigation based on multidimensional bioinformatics and molecular simulation

**DOI:** 10.1371/journal.pone.0347532

**Published:** 2026-04-20

**Authors:** Changze Ou, Binbin Chen, Jun Deng, Huajun Long

**Affiliations:** 1 Graduate School, Hunan University of Chinese Medicine, Changsha, Hunan, China; 2 Department of Emergency, Hunan Provincial Hospital of Integrated Traditional Chinese and Western Medicine (Affiliated Hospital of Hunan Academy of Traditional Chinese Medicine), Changsha, Hunan, China; 3 Department of Neurology, Hunan Provincial Hospital of Integrated Traditional Chinese and Western Medicine (Affiliated Hospital of Hunan Academy of Traditional Chinese Medicine), Changsha, Hunan, China; Shiga Medical Center, JAPAN

## Abstract

**Background:**

Histone deacetylases (HDACs) regulate neuroprotection; however, Trichostatin A (TSA), an HDAC inhibitor, lacks clear molecular mechanisms and core targets in Alzheimer’s disease (AD), limiting clinical translation. This study aimed to decipher TSA’s AD-regulating network, screen core genes, and support AD early diagnosis and multi-target therapies.

**Methods:**

TSA targets were computationally predicted. Five GEO AD datasets were analyzed for differential genes and core modules, and 130 machine learning algorithms were employed to identify core genes. Functional annotation, immune cell analysis, and single-cell expression profiling were conducted. Molecular docking and 100 ns molecular dynamics simulations verified TSA-protein interactions.

**Results:**

949 potential TSA targets were identified, overlapping with AD differential genes and enriching key pathways such as GABAergic synapse and tau phosphorylation. Eight machine learning-identified core genes (EFNA1, GABRB2, GABARAPL1, EGR1, CDK5, KCNC2, MET, GRIA2) exhibited a distinct AD expression pattern: synergistic downregulation of protective genes and unique upregulation of pathological EFNA1. These genes are implicated in neurotransmission, synaptic plasticity, tau clearance, and immune-neural crosstalk. Molecular dynamics simulations suggested TSA may not stably bind these candidates, implying its regulation relies on epigenetic mechanisms via HDAC1–3/6 inhibition, potentially restoring gene network balance and disrupting neuroinflammation-neurodegeneration cycles. Complex regulatory modes and cell type-specific expression were also observed.

**Conclusion:**

This study provides preliminary insights into TSA’s putative mechanisms in AD intervention, highlighting the eight candidate core genes’ potential diagnostic and therapeutic value as AD biomarkers, supporting TSA’s multi-target therapy. All findings are computationally derived and require experimental verification.

## 1. Introduction

Histone Deacetylases (HDACs) are a class of key enzymes that primarily remove acetyl modifications from histones through deacetylation, thereby regulating gene expression [1]. They play critical roles in cell proliferation, differentiation, apoptosis, and also target non-histones to regulate neuroprotection, metabolism, and inflammation [2]. HDACs are classified into four classes: Class I/II (zinc-dependent, nuclear histone deacetylation), Class III (Sirtuins, NAD⁺-dependent, energy metabolism/homeostasis), and Class IV (limited research, specific functions) [3]. HDAC inhibitors (HDACi) selectively or non-selectively inhibit HDAC activity, enhancing histone/non-histone acetylation to activate gene transcription and regulate cell fate [4]. This class of drugs has been widely used in tumor treatment and has gradually shown potential clinical value in fields such as cardiovascular diseases and neurodegenerative diseases [5].

In the field of neurological diseases, especially in the research of Alzheimer’s Disease (AD), the role of HDACs and their inhibitors has attracted great attention [6]. AD is a neurodegenerative disease characterized by the gradual decline of cognitive function, and its typical pathological manifestations include the deposition of amyloid-β(Aβ) plaques, neurofibrillary tangles formed by the excessive phosphorylation of tau protein, and neuronal loss [7]. Studies have found that HDACs play an important regulatory role in the occurrence and development of AD, especially in regulating the deposition of Aβ in the mouse brain, the survival and death of neurons, and inflammatory responses [8]. By regulating the expression of genes related to amyloid-β protein precursor (AβPP) and Aβ-converting enzyme 1 (BACE1), HDACs affect the metabolic pathway and clearance mechanism of amyloid protein, thereby participating in the disease process [9]. In animal models of AD, the HDAC inhibitor Trichostatin A (TSA) has exhibited significant therapeutic effects, including improving cognitive function, promoting neuroregeneration, reducing neuroinflammatory responses in the brain, and attenuating the pathological accumulation of Aβ and tau proteins [10]. Some studies have pointed out that HDAC inhibitors can also activate the expression of brain-derived neurotrophic factor (BDNF) and other factors by regulating multiple signaling pathways related to neurodegenerative diseases, thereby enhancing neural plasticity and cognitive ability [11].

However, although existing studies have shown that HDAC inhibitors have potential therapeutic advantages, their specific mechanism of action in regulating AD-related molecular networks is not yet fully clear. Currently, most existing studies focus on the regulation of TSA on a single target (such as HDAC1) or a single pathology (such as Aβ deposition), which limits the comprehensive understanding of its molecular network regulating AD [12]. To sum up, the study on the molecular network of TSA regulating Alzheimer’s Disease has important medical and scientific significance. Against this background, this study aims to systematically analyze the molecular network of TSA regulating AD through multi-dimensional bioinformatics analysis, machine learning modeling, and molecular simulation technology, screen key core genes and intervention targets, and provide a theoretical basis for the early diagnosis of AD and multi-target therapeutic strategies.

## 2. Methods

### 2.1. Acquisition of chemical composition and genes of TSA

**Fig 1:**
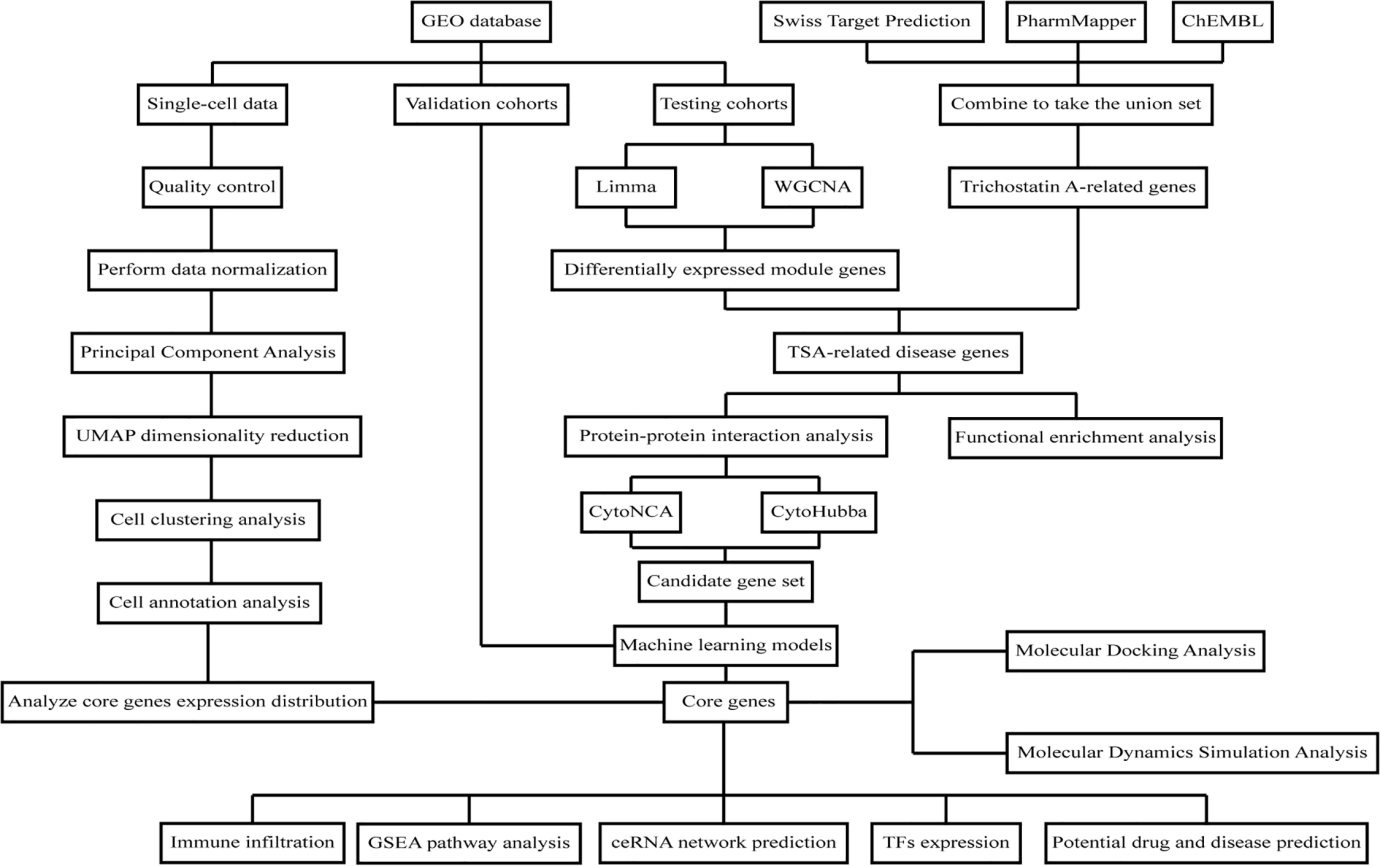
Flow-chart of dataset analysis in this paper. This workflow delineates the regulatory network of TSA in AD, encompassing TSA putative target prediction, AD-related gene and core module screening from 5 GEO datasets, core gene identification via 130 integrated machine learning algorithms, multi-dimensional functional validation (including immune and single-cell profiling, regulatory network analysis), and ligand-receptor binding verification via molecular docking and 100 ns molecular dynamics simulations.

To clarify the functional basis of TSA, multi-database resources were integrated for characteristic analysis and target prediction. The physicochemical properties and biological activity parameters of TSA were retrieved from PubMed, and its standard two-dimensional structural descriptor (SMILES: C[C@H](/C = C(\C)/C = C/C(=O)NO)C(=O)C1 = CC = C(C = C1)N(C)C)) was extracted from PubChem (https://pubchem.ncbi.nlm.nih.gov/) [13]. Potential targets of the human proteome were predicted via a triple strategy: ligand-receptor interaction analysis (ChEMBL, https://www.ebi.ac.uk/chembl/) [14], chemogenomic prediction (SwissTargetPrediction, http://www.swisstargetprediction.ch) [15], and three-dimensional pharmacophore matching (PharmMapper, http://lilab-ecust.cn/pharmmapper) [16]. Overlap and integration of the resulting gene sets were analyzed and visualized in R, with the complete analytical workflow presented in Fig 1.

### 2.2. Collection and preprocessing of AD-related datasets

To explore AD-related gene expression characteristics, five Homo sapiens datasets were retrieved from the Gene Expression Omnibus (GEO) database (https://www.ncbi.nlm.nih.gov/geo/) (keyword: ‘Alzheimer’s disease’): GSE122063/GSE132903 for training (model construction), GSE44771/GSE109887 for validation (performance evaluation), and GSE161045 for single-cell analysis (Table 1). Training data underwent sequential processing: data transformation, duplicate averaging, log2 transformation, and cross-chip normalization. Matched control/AD samples were extracted, labeled, merged with gene expression data, and common genes integrated. Batch effects were corrected via the ComBat algorithm, with effectiveness assessed by pre-/post-correction PCA scatter plot visualization.

**Table 1:** Dataset content.

GSE series	Samples	Platform	Data type
GSE122063	56 AD patients and 44 healthy controls	GPL16699	Training cohort
GSE132903	97 AD patients and 98 healthy controls	GPL10558	Training cohort
GSE44771	129 AD patients and 101 healthy controls	GPL4372	Validation cohort
GSE109887	46 AD patients and 32 healthy controls	GPL10904	Validation cohort
GSE161045	4 AD patients and 4 healthy controls	GPL24676	Sing-cell data

### 2.3. Acquisition of AD-related genes

Limma calculated log2 fold change (logFC) and adjusted P-value (adj.P.Val) for the batch-corrected training set expression matrix; DEGs were screened with |logFC| > 0.4 and adj.P.Val < 0.05 (per reviewer comment) and visualized via pheatmap heatmaps, ggplot2 volcano plots and PCA scatter plots. WGCNA was used to construct a gene co-expression network: post sample quality control, modules were clustered by topological overlap matrix (TOM), key modules identified by correlating module eigengenes with clinical traits, and module clustering trees, trait correlation heatmaps and module membership-gene significance scatter plots used for interpretation, with a comprehensive result table generated.

### 2.4. Identification of TSA-related disease genes

Intersection analysis was performed on DEGs, WGCNA core genes, and TSA-predicted genes to identify the TSA-related disease gene set, and the intersection results were visualized using a Venn diagram to clarify the scope of core genes related to the mechanism of TSA action and the pathophysiological process of AD.

### 2.5. Functional and pathway enrichment analysis based on KEGG, DO, and GO databases

Gene symbols were standardized to Entrez Gene IDs for accuracy. We performed enrichment analyses for KEGG pathways (metabolism/signal transduction), Disease Ontology (DO) (gene-disease associations), and Gene Ontology (GO) (molecular function, cellular component, biological process). Significant results were filtered with thresholds of p < 0.05 and q < 0.05, and visualized as required.

### 2.6. Construction of PPI Network

A PPI network was constructed using data from the STRING database (https://string-db.org/) (confidence threshold = 0.400, isolated nodes hidden) [17], and visualized with Cytoscape 3.10.1. Key nodes were screened with cytoHubba and CytoNCA plugins, and the intersection of the top 20 genes from both plugins was defined as the candidate core gene set for subsequent machine learning.

### 2.7. Core gene screening based on 130 machine learning algorithms

Data preprocessing: missing values and outliers were removed to ensure authenticity; common genes from training/validation sets filtered redundant features; Z-score standardization (mean = 0, standard deviation = 1) eliminated dimensional differences; batch effects were corrected by cohort; low-variance Gaussian noise (sd = 0.01) enhanced clinical anti-interference ability; data were randomly split into training/test sets at a 7:3 ratio (categorical variables: AD patients/healthy controls; feature/label matrices defined). A two-stage “variable selection-model training” strategy was applied to construct 130 machine learning models with 11 core algorithms: the variable selection stage integrated 9 classic linear, nonlinear and ensemble algorithms (Elastic Net, Lasso, Ridge, Stepwise GLM, SVM, LDA, GBM, RF, XGBoost, Naive Bayes) for multi-algorithm cross-validation, extracting high-importance features by removing invalid ones with the minimum retained variable threshold; the model training stage built individual models with screened features, optimized hyperparameters via 5/10-fold cross-validation, integrated valid models (≥2 variables) into the final set by logistic regression, and used a fixed random seed throughout for reproducibility. All core algorithms adopted unified optimized hyperparameters for reliable model comparison: Elastic Net (λ = 0.1, alpha tunable), Lasso (λ = 0.05, alpha = 1) and Ridge (λ = 1.0, alpha = 0) with optimal λ selected by 10-fold cross-validation; SVM (C = 1.0, γ = 0.01); RF (ntree = 200, nodesize = 5), with feature importance and proximity calculated during modeling; XGBoost (max.depth = 2, learning rate = 0.01, n.trees = 150), optimal iterations determined by 5-fold cross-validation and objective function set to binary:logistic; GBM (n.trees = 100, learning rate = 0.1, interaction.depth = 3, n.minobsinnode = 10), optimal iterations selected by 10-fold cross-validation; glmBoost with mstop optimized via k-fold cross-validation (minimum mstop = 40); Stepwise GLM, LDA, Naive Bayes and plsRglm used default optimal parameters of their respective R packages. All above algorithms took binary classification as the core modeling objective. Performance evaluation: Area Under the Receiver Operating Characteristic Curve (AUROC) was the core indicator (clinical effectiveness threshold: 0.7); the RunEval function calculated cohort-stratified AUCs for training/test sets, and heatmaps visualized model performance differences. Feature importance was extracted to identify the top 10 core genes (key gene-model importance heatmap verification), and the model with the highest AUC was selected as the optimal diagnostic model (generalization ability and clinical potential systematically evaluated).

### 2.8. Core gene performance evaluation and expression difference analysis

Pearson correlation analysis, performed on preprocessed (transposed, standardized) gene expression data, clarified correlations among the 8 core AD target genes. A comprehensive chart displayed these relationships, including histograms, kernel density curves, confidence ellipses, scatter plots, smooth trend lines, correlation coefficients (3 decimal places), and significance stars. A Logistic regression model was constructed based on optimal model core genes, with a nomogram for individualized risk assessment. Calibration curves evaluated prediction accuracy, decision curve analysis (DCA) assessed clinical net benefit, and stacked bar charts displayed high-risk cases, sensitivity, and cost-benefit ratio. ROC curves (combined model/single genes) and AUC with confidence intervals evaluated diagnostic efficacy. The SHAP method (kernelshap package) analyzed the model (shapviz package visualized importance bar charts, bee swarm plots, etc.) to identify core genes and their impact on diagnosis. The DALEX package performed feature permutation importance analysis. For each core gene, t-tests evaluated inter-group expression differences (box plot visualization), and ROC curves/AUCs assessed individual diagnostic efficacy.

### 2.9. Quantitative analysis of immune infiltration

Reference and mixed sample gene expression profiles were preprocessed (standardization, gene screening) for quality control. Support vector regression (SVR) with quantitative normalization quantified immune cell proportions (grouped by disease); significantly infiltrated cells were identified by permutation tests (P < 0.05). Visualization employed stacked bar charts, box plots, correlation heatmaps, and ridge plots. Spearman correlation assessed associations between core genes and immune cell infiltration (P-values calculated), presented in gene-immune cell and immune cell-immune cell correlation heatmaps.

### 2.10. GSEA pathway analysis of core genes

Samples were divided into high/low-expression groups by core gene expression. logFC was calculated, and Gene Set Enrichment Analysis (GSEA) evaluated associations with predefined KEGG pathways. Significantly enriched up/down-regulated pathways were visualized for each core gene.

### 2.11. Construction of Competitive Endogenous RNA (ceRNA) network

MiRNAs targeting core genes were identified by integrating predictions from miRDB, TargetScan, and miRanda, selecting those common to all three databases. Subsequently, spongeScan identified lncRNAs binding these miRNAs. The resulting lncRNA-miRNA network was visualized using Cytoscape (v3.10.1), elucidating the lncRNA-miRNA-mRNA regulatory relationship.

### 2.12. Systematic identification of the master regulatory transcription factor network of gene sets

The RcisTarget package (hg19 motif ranking database) performed motif enrichment analysis, evaluating the weighted AUC of the target gene set under each motif. Significantly enriched motifs were screened with normalized enrichment score (NES) > 3.5, and annotated to corresponding transcription factors. Cumulative recovery curves verified target gene enrichment, the igraph package constructed a transcription factor-target gene bipartite network, and visNetwork/networkD3 generated interactive network graphs and Sankey diagrams. Significantly enriched motifs and regulatory information were integrated into an interactive HTML table.

### 2.13. Single-cell data analysis

Data preprocessing: Seurat constructed single-cell objects (filtering criteria: genes expressed in ≥5 cells, cells with ≥100 genes); quality control eliminated cells with <300 genes or mitochondrial gene proportion >20%. Data were normalized via LogNormalize (normalization factor = 10,000), and variance stabilizing transformation (VST) identified the top 2,500 highly variable genes. Dimensionality reduction: PCA (22 principal components), K-nearest neighbor graph (first 15 PCs, resolution = 0.6), UMAP visualization (first 15 PCs). Differential gene analysis (logFC ≥ 1.0, adj.P.Val < 0.05, limma package) identified cluster marker genes. Clusters were annotated via comparison with known cell type marker genes and enrichment score matrix analysis (heatmap, DotPlot visualization). Core gene expression was visualized via FeaturePlot and VlnPlot; average expression matrices, cell type annotations, and merged multi-gene DotPlots/VlnPlots were output.

### 2.14. Molecular docking analysis and molecular dynamics simulation analysis

TSA structure (SDF format) and core target 3D structures were obtained from PubChem and UniProt (https://www.uniprot.org/), respectively. Molecular docking was performed using CB-Dock2 (https://cadd.labshare.cn/cb-dock2) with AutoDock Vina (v1.1.2), incorporating CurPocket cavity detection and FitDock template-based docking. Proteins were preprocessed (missing side chains and hydrogens supplemented), and ligands were prepared with hydrogens and partial charges. Homologous templates were screened from BioLip using FP2 fingerprints (similarity ≥ 0.4) and BLASTP (E-value < 1e-5). Conformations with Root Mean Square Deviation (RMSD) < 2.0 Å were considered valid [18,19]. Molecular dynamics (MD) simulations of the top 2 ligand-target complexes were conducted using GROMACS 2023.2 (Ubuntu 22.04). Ligands were processed with GAFF (SOBTOP), and proteins with Amber99sb-ildn force fields. Systems were solvated in TIP3P water with Na ⁺ /Cl⁻ for neutrality, followed by energy minimization, 100 ps NVT (300 K), 100 ps NPT (1 bar), and 100 ns production run (2 fs timestep). Long-range electrostatics were computed via PME; bond lengths constrained via LINCS. RMSD, Root Mean Square Fluctuation (RMSF), and hydrogen bond dynamics were analyzed using GROMACS tools; binding energy was calculated via MMPBSA (APBS 3.4.1).

### 2.15. Disease and drug prediction

Eight core genes were input into the DisGeNET database (https://disgenet.com/), which integrates data from public databases, GWAS catalogs, animal models, and scientific literature) to predict diseases related to the core genes [20]. The DSigDB database (https://dsigdb.tanlab.org/DSigDBv1.0/) was used to predict candidate drugs related to specific genes, and the P-value corrected by the false discovery rate (FDR) was set to less than 0.05 to reduce the false positive of multiple tests, and finally 15 drugs associated with the core genes were displayed [21].

## 3. Results

### 3.1. Identification of TSA target proteins

**Fig 2:**
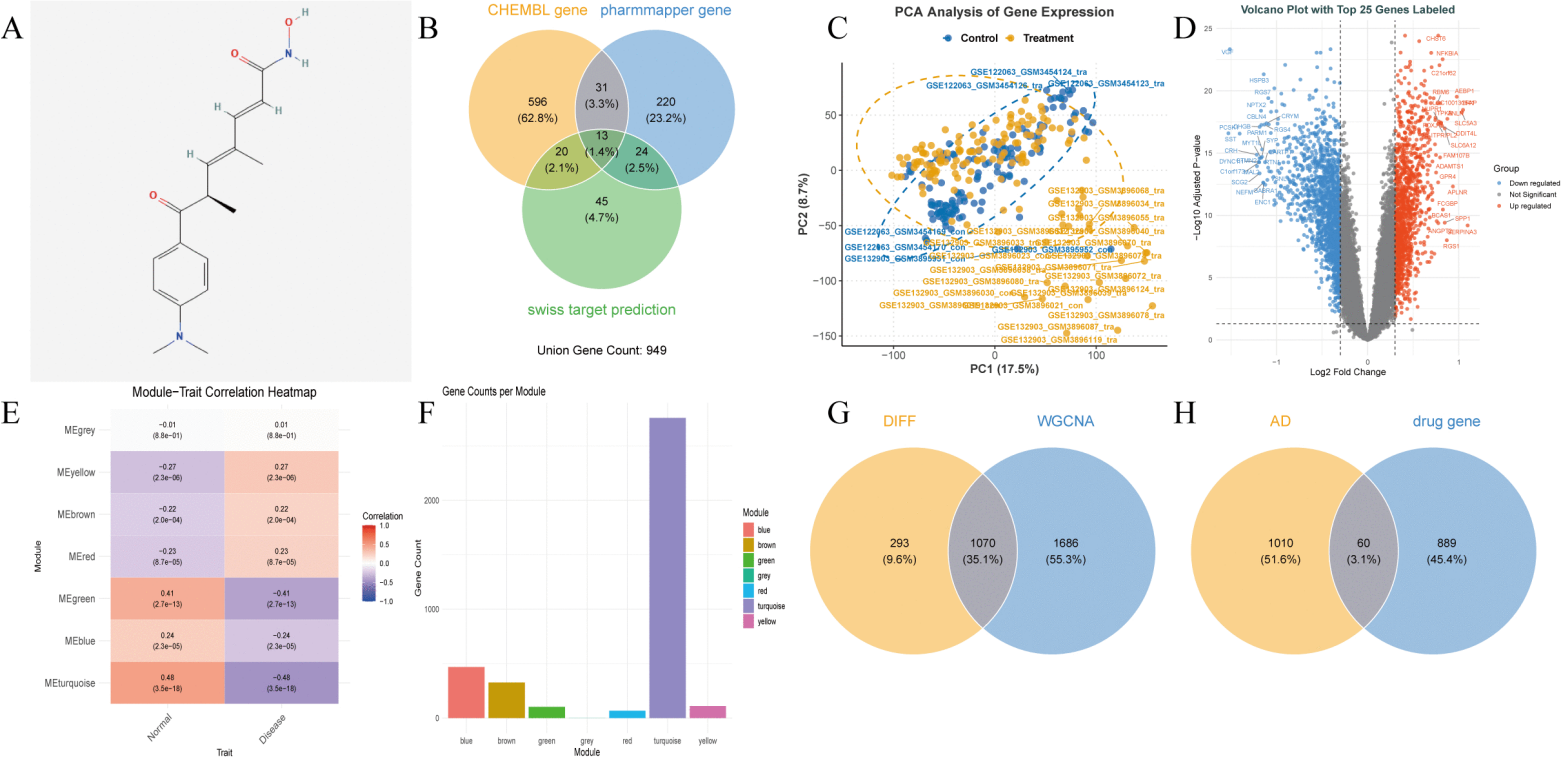
Schematic of TSA target prediction and AD-related gene screening. **(A)**. 2D chemical structure of TSA (PubChem CID: 444732). **(B)**. Venn diagram of TSA potential targets predicted by ChEMBL, PharmMapper and SwissTargetPrediction, with 949 non-redundant unique targets obtained. **(C)**. PCA plots of merged training cohort (n = 295) before/after ComBat batch correction. **(D)**. Volcano plot of AD DEGs (screening thresholds: |logFC| > 0.4, adj.P.Val < 0.05). **(E)**. Heatmap of module-trait correlations from WGCNA. **(F)**. Bar chart of gene counts in each WGCNA co-expression module. **(G)**. Venn diagram of the intersection between 1363 DEGs and 2756 genes in the WGCNA MEturquoise module (1070 overlapping genes obtained). **(H)**. Venn diagram of the intersection between 1070 module genes and 949 TSA targets (60 key genes screened).

The molecular structure of TSA was obtained from the PubChem database (Fig 2A). Potential biological targets of TSA were predicted using three distinct databases: ChEMBL (661 targets), PharmMapper (290 targets), and SwissTargetPrediction (102 targets). After data integration and redundancy removal, a total of 949 unique potential targets of TSA were identified (Fig 2B) (S1 Table).

### 3.2. Screening and identification of potential target genes for AD

PCA of the training set data revealed significant batch effects, which were eliminated by the ComBat algorithm (effective clustering of AD samples post-correction, Fig 2C). Differential expression analysis with thresholds |logFC| > 0.4 and adj.P.Val < 0.05 identified 1363 DEGs: 814 down-regulated (59.7%) and 549 up-regulated (40.3%). A volcano plot was generated to display the top 25 genes with the highest logFC values among the up-regulated and down-regulated genes (Fig 2D). WGCNA identified 7 gene modules (Fig 2E), among which the MEturquoise module (2756 genes) showed the strongest correlation with AD (r = 0.48, Fig 2F). Intersection of DEGs and MEturquoise module genes yielded 1496 differential module genes. Further intersection with TSA potential targets identified 60 TSA-related AD key genes (Fig 2G,H), putatively involved in TSA’s mechanism and AD pathophysiology (S2 Table).

### 3.3. Identification and analysis of TSA-related disease targets in AD

**Fig 3:**
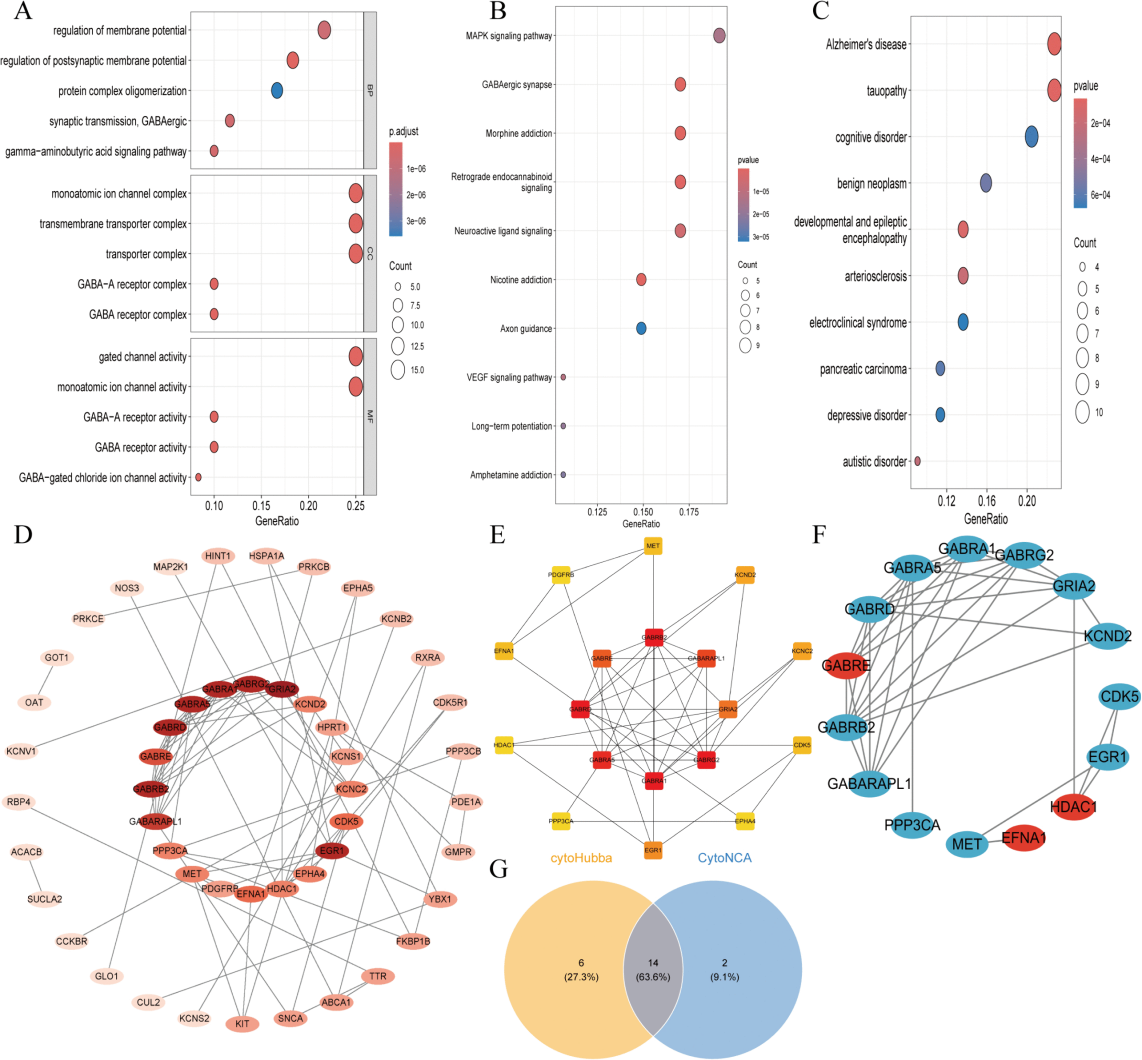
Functional Annotation of 60 TSA-associated Key Genes for AD and PPI Network Analysis. **(A-C)**. GO, KEGG, and DO enrichment bubble plots (significance threshold: P < 0.05, adjusted q < 0.05). **(D)**. PPI network constructed based on the STRING database (confidence threshold was set at 0.4). **(E)**. Key nodes of the PPI network identified by the cytoHubba plugin. **(F)**. Key PPI nodes identified by CytoNCA plugin; red = upregulated genes in AD, blue = downregulated genes in AD. **(G)**. Venn diagram of the intersection of screening results from cytoHubba and CytoNCA (a total of 14 candidate core genes were obtained).

Functional enrichment analysis of the 60 key genes was performed via KEGG, GO, and DO databases: GO enrichment was focused on GABAergic synaptic transmission (biological process), GABA-A receptor complexes (cellular component), and GABA receptor activity (molecular function) (Fig 3A); KEGG pathway enrichment: 77 significantly enriched pathways, with core AD-related pathways focused on GABAergic synapse and neuroactive ligand-receptor interaction (Fig 3B); DO enrichment: Associated with AD, tauopathies, and cognitive impairment (Fig 3C). A protein-protein interaction (PPI) network was constructed via Cytoscape (Fig 3D). Intersecting the hub genes from cytoHubba (Fig 3E) and CytoNCA (Fig 3F) analyses identified 14 candidate core genes: EFNA1, GABRA5, GABRD, HDAC1, GABRB2, GABARAPL1, EPHA4, EGR1, CDK5, PPP3CA, KCNC2, MET, KCND2, and GRIA2 (Fig 3G) (S3 Table).

### 3.4. Core gene screening via model construction based on 130 machine learning algorithms

**Fig 4:**
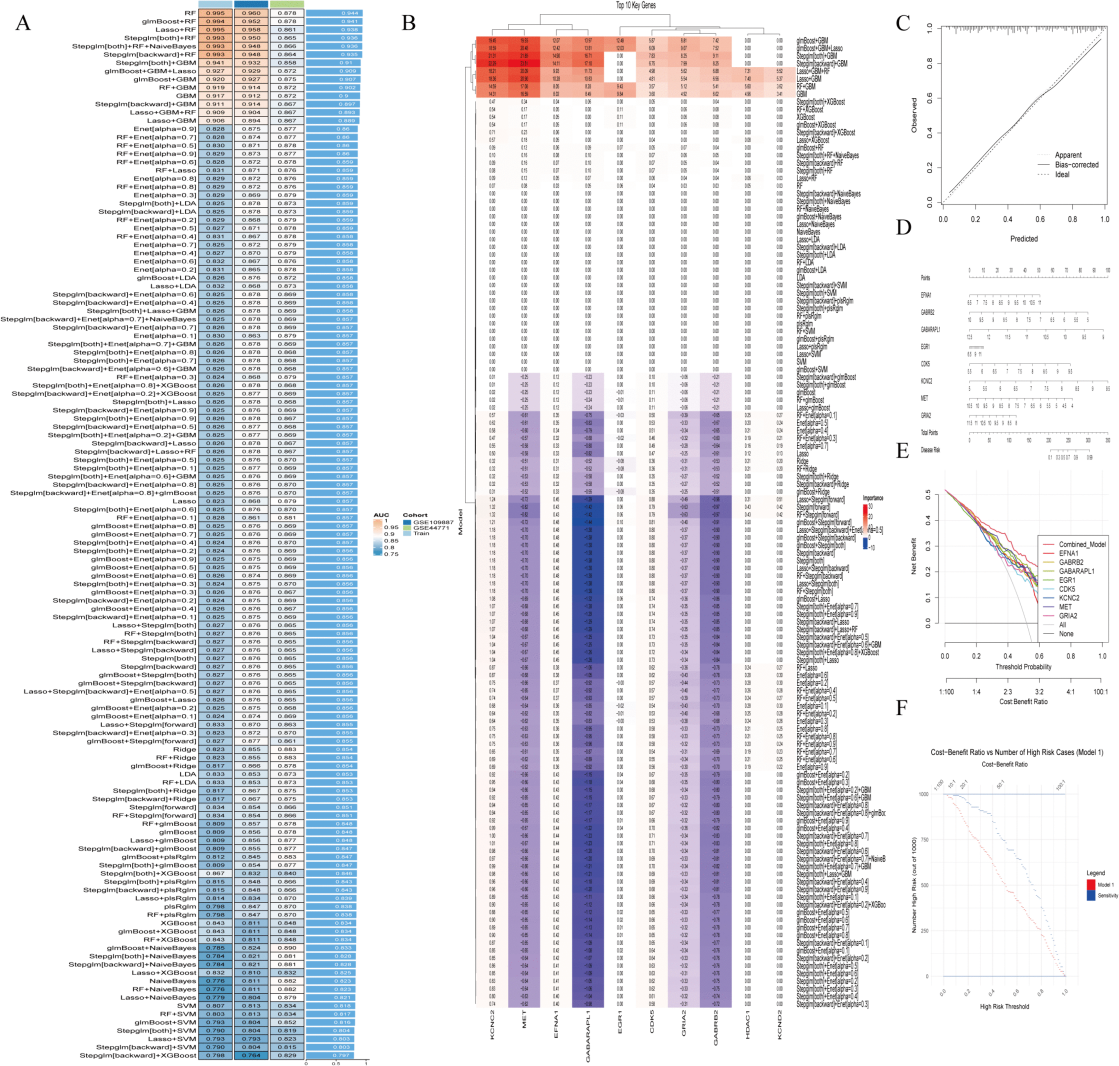
Construction and Performance Evaluation of Machine Learning Models for Core Gene Screening. **(A)**. AUROC heatmap of 130 models across 3 cohorts, with the optimal glmBoost + RF model (AUROC = 0.994) marked in red, used for final core gene screening and AD risk prediction. **(B)**. Top 10 gene feature importance bar plot of the optimal model, quantifying each gene’s contribution to AD diagnostic efficacy. **(C)**. Calibration curve of the optimal model (45° line = perfect calibration), verifying the consistency between predicted and actual AD risk. **(D)**. Nomogram for individualized AD risk prediction based on the optimal model, quantifying the independent contribution of each core gene to AD risk. **(E)**. DCA plot comparing clinical net benefit of the combined model vs. single-gene prediction, confirming the superior clinical application value of the 8-gene model. **(F)**. Cost-benefit analysis plot based on the GSE44771 dataset, evaluating the model’s clinical application potential under different risk thresholds.

**Fig 5:**
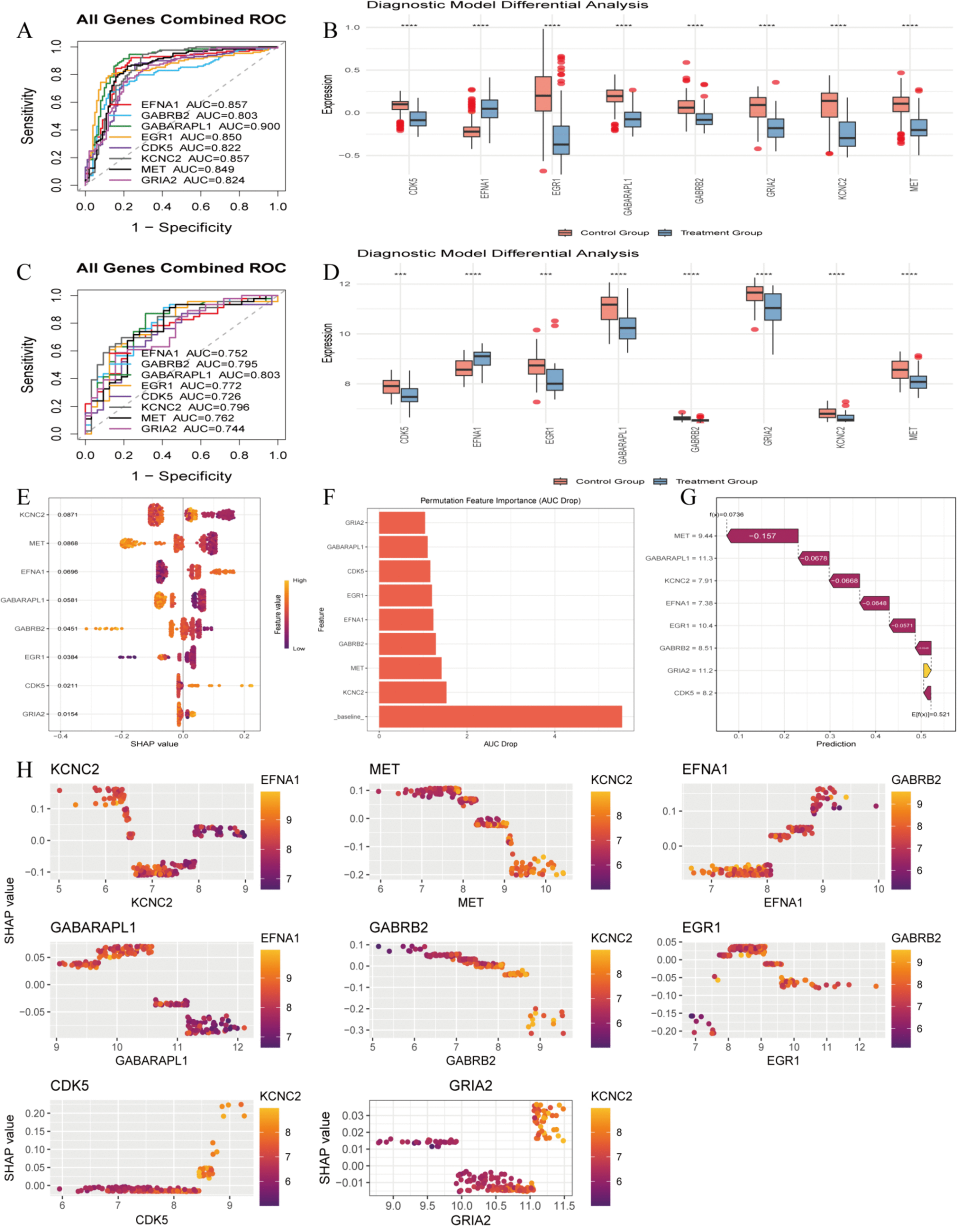
Diagnostic efficacy validation of 8 core genes and SHAP-based model interpretability analysis. **(A-C)**. ROC curves of the 8-gene combined model in GSE44771 (129 AD, 101 controls) and GSE109887 (46 AD, 32 controls) validation cohorts, verifying the stable diagnostic performance of the core genes. **(B-D)**. Box plots of 8 core genes’ expression in two validation cohorts; unpaired Student’s t-test, **** P < 0.0001, showing the significant AD-specific differential expression pattern of core genes. **(E)**. SHAP beeswarm plot of core genes ranked by mean SHAP value, clarifying the direction and magnitude of each gene’s effect on AD prediction. **(F)**. Bar plot of AUC reduction after gene permutation, identifying KCNC2 as the top contributor to the model’s predictive performance. **(G)**. SHAP waterfall plot for a representative AD sample, intuitively showing each gene’s contribution to individual AD risk prediction. **(H)**. SHAP scatter plot of core genes, revealing the nonlinear effect of core genes on AD risk and their co-expression pattern.

130 machine learning algorithms were used to construct prediction models, with performance visualized by heatmaps (Fig 4A) and top 10 gene feature importance plots (Fig 4B). The optimal model was the glmBoost + RF combination (AUC = 0.994), screening 8 core genes: EFNA1, GABRB2, GABARAPL1, EGR1, CDK5, KCNC2, MET, GRIA2. Model validation confirmed its good predictive performance: calibration curve verified accuracy (Fig 4C), nomogram showed gene contribution weights (Fig 4D), DCA demonstrated superior clinical net benefit over individual genes (Fig 4E), and cost-benefit analysis confirmed robustness (Fig 4F). In independent validation sets GSE44771 (AUC > 0.80) and GSE109887 (AUC > 0.72), core genes exhibited stable diagnostic performance. Expression pattern analysis revealed EFNA1 was significantly up-regulated in AD, while other core genes were down-regulated (Fig 5A-D). SHAP analysis indicated core gene low expression was significantly associated with AD (positive SHAP values, Fig 5E), with KCNC2 contributing most to model accuracy (Fig 5F). A force plot visualized single-sample prediction (Fig 5G), and core genes showed characteristic nonlinear effects (e.g., KCNC2, GRIA2) and co-expression patterns (EFNA1 high expression accompanied by low expression of other cores, Fig 5H). These genes are implicated in key AD pathological processes (abnormal GABAergic neurotransmission, impaired synaptic plasticity)(S4 Table). Pearson correlation analysis was performed for the 8 core AD target genes using the psych package in R, and the results showed extremely high statistical significance (***, P < 0.001). GABARAPL1 and GABRB2 (r = 0.882), as well as GABARAPL1 and CDK5 (r = 0.834), exhibited extremely strong positive correlations. The 7 genes including GABRB2 and CDK5 were predominantly positively correlated with each other, while EFNA1 showed significant negative correlations with all other core genes (the strongest correlation was with GRIA2, r = −0.624). This correlation pattern provides crucial data support for deciphering the molecular regulatory network of AD and developing multi-target intervention strategies (S1 Fig).

### 3.5. Correlation analysis between immune infiltration characteristics and core gene expression

**Fig 6:**
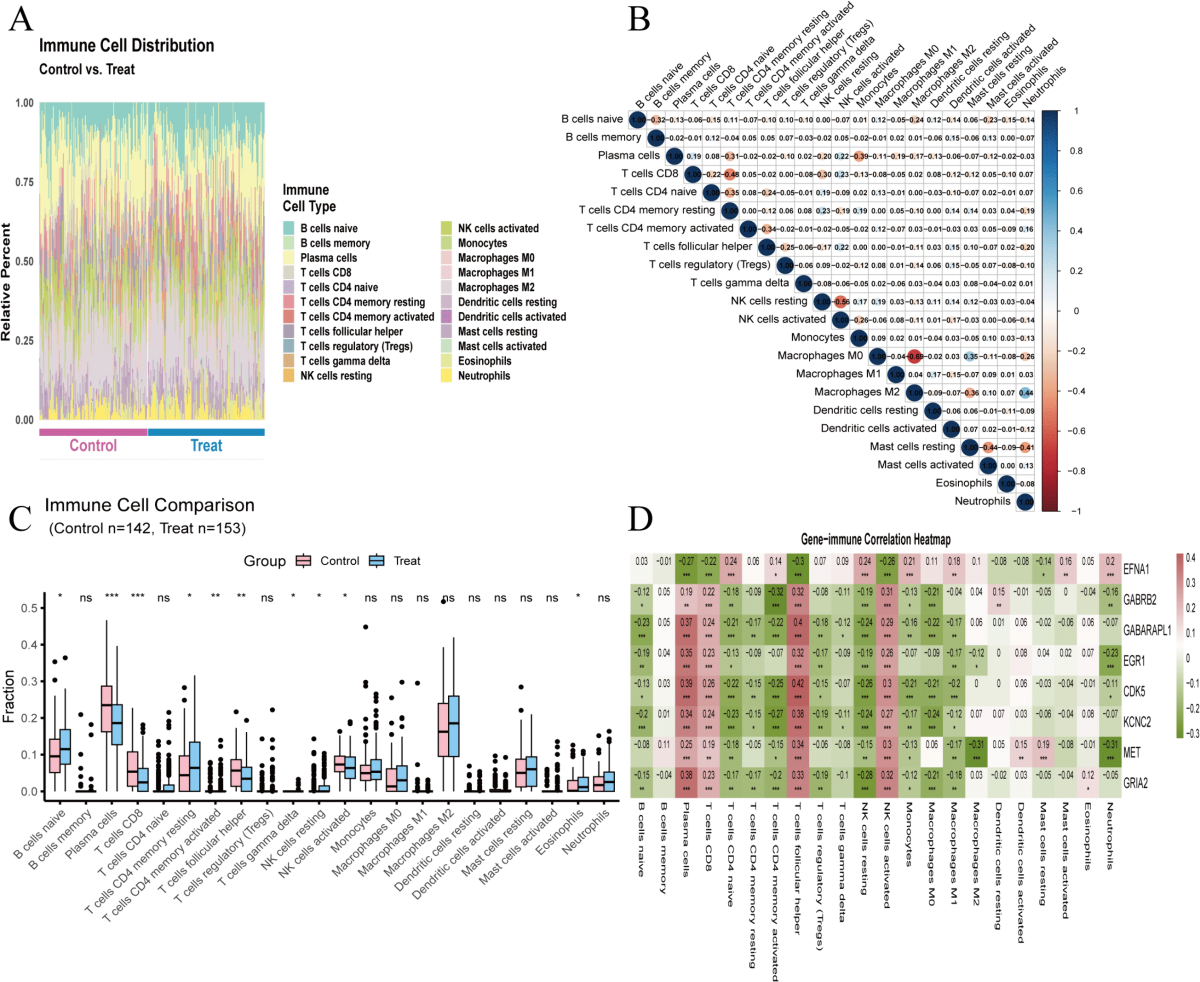
Immune infiltration characteristics of AD and correlation with 8 core genes analyzed via CIBERSORT. **(A)**. Immune cell abundance heatmap of the training set (142 Ctrl, 153 AD cases). **(B)**. Spearman correlation heatmap of infiltrating immune cell populations. **(C)**. Box plot of differentially infiltrated immune cells between AD and Ctrl groups (* P < 0.05, *** P < 0.001). **(D)**. Spearman correlation heatmap between core genes and infiltrating immune cells, significant results (adjusted P < 0.05) marked in bold.

Immune cell infiltration was analyzed via the optimized CIBERSORT algorithm (1000 permutation tests, P < 0.05). The AD immune microenvironment was mainly characterized by Plasma cells, M2 Macrophages, naive B cells and CD8 + T cells (Fig 6A), with significant pairwise correlations between immune subsets (Fig 6B) and differential infiltration of Plasma cells, CD8 + T cells and activated CD4 + memory T cells between groups (Fig 6C). EFNA1, the only AD-upregulated core gene, showed an immune correlation pattern opposite to other downregulated core genes, and was significantly associated with multiple AD-related immune subsets (Fig 6D). These results revealed AD-specific immune microenvironment alterations and the unique immune correlation of EFNA1, providing clues for AD immunopathology research and immunomodulatory therapy (S5 Table).

### 3.6. Results of core gene GSEA Analysis, ceRNA network, and TFs analysis

**Fig 7:**
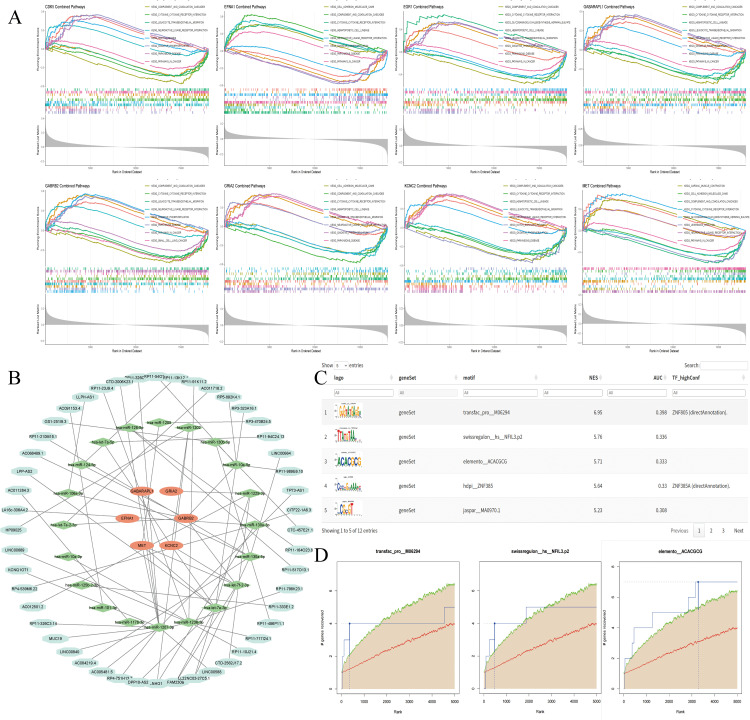
Pathway Enrichment Analysis of Core Genes and Regulatory Network Construction. **(A)**. GSEA enrichment plot of core genes, highlighting top AD-related significant pathways. **(B)**. ceRNA regulatory network centered on 8 core genes (48 lncRNAs, 51 miRNAs; light green = lncRNAs, green = miRNAs, orange = core mRNAs). **(C)**. Bar chart of NES of significantly enriched TF motifs (RcisTarget, NES > 3.5). **(D)**. TF motif gene recovery curve, verifying the enrichment reliability of core target genes.

GSEA showed core genes were significantly enriched in AD-related pathways, including Complement and Coagulation Cascades, Neuroactive Ligand-Receptor Interaction, and Oxidative Phosphorylation (Fig 7A). A ceRNA regulatory network centered on core genes was constructed, involving 51 miRNAs and 48 lncRNAs (Fig 7B). Transcription factor (TF) analysis identified ZNF805 and ZNF385A as master regulatory factors (Fig 7C,D), with characteristic binding motifs visualized. These results reveal multi-level regulatory mechanisms (transcriptional, post-transcriptional) of core genes in AD (S6 Table).

### 3.7. Single-cell data UMAP dimensionality reduction and cell annotation

**Fig 8:**
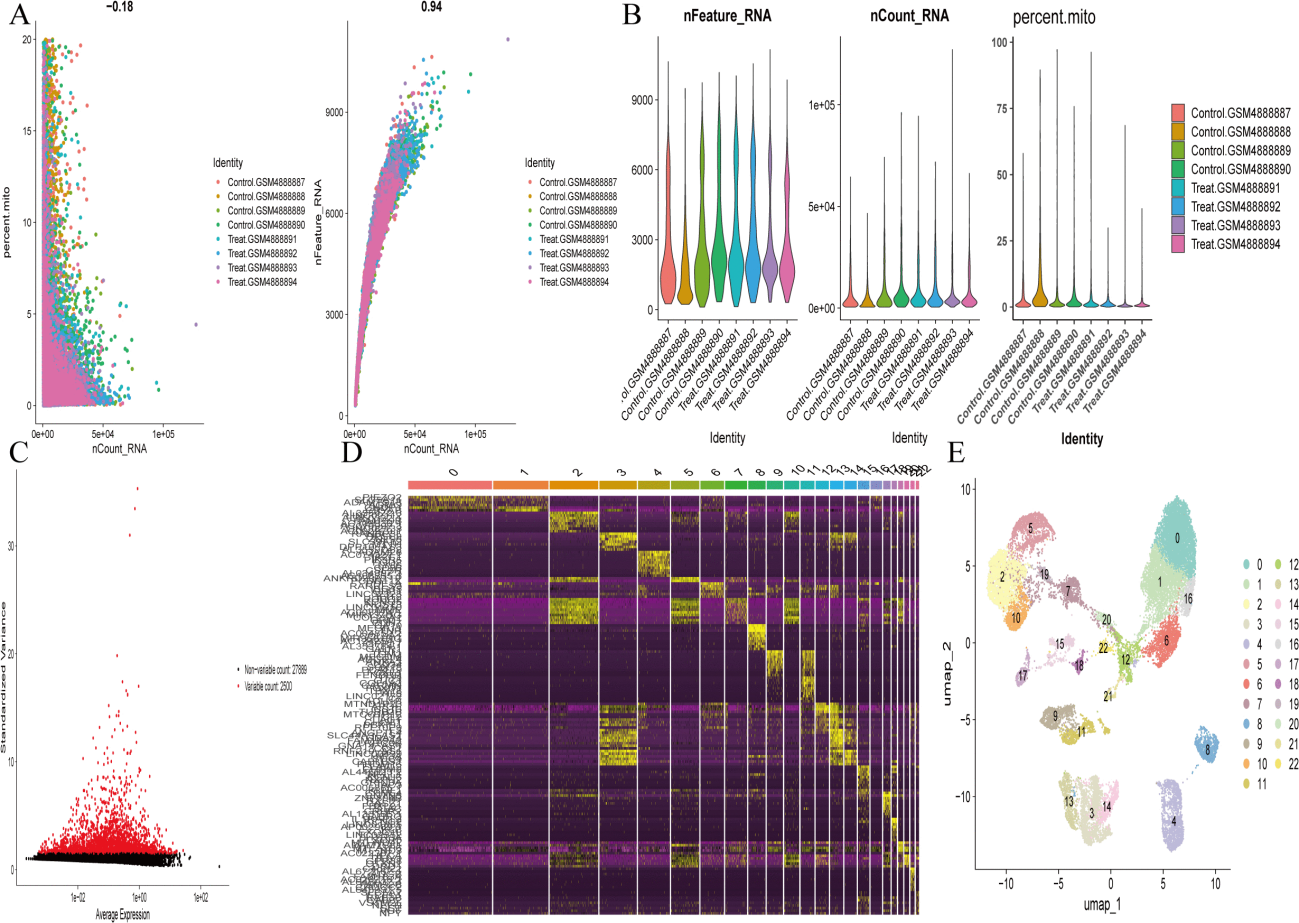
Preprocessing and dimensionality reduction analysis of scRNA-seq data (GSE161045: 4 AD vs 4 controls) performed via Seurat. **(A)**. PCA plot of scRNA-seq samples, showing cell population separation between AD and Ctrl groups. **(B)**. Quality control distribution plot (filtering criteria: ≥ 300 genes/cell, mitochondrial ratio <20%), ensuring high data quality for subsequent analysis, ensuring high data quality for subsequent analysis. **(C)**. Scatter plot of gene average expression vs. normalized variance; 2500 highly variable genes are marked in red, providing the basis for cell clustering and annotation. **(D)**. Heatmap of cluster-specific marker genes, showing the unique expression signature of each cell cluster. **(E)**. UMAP plot of 22 cell clusters (resolution = 0.6, top 15 PCs), displaying the spatial distribution of brain cell populations.

**Fig 9:**
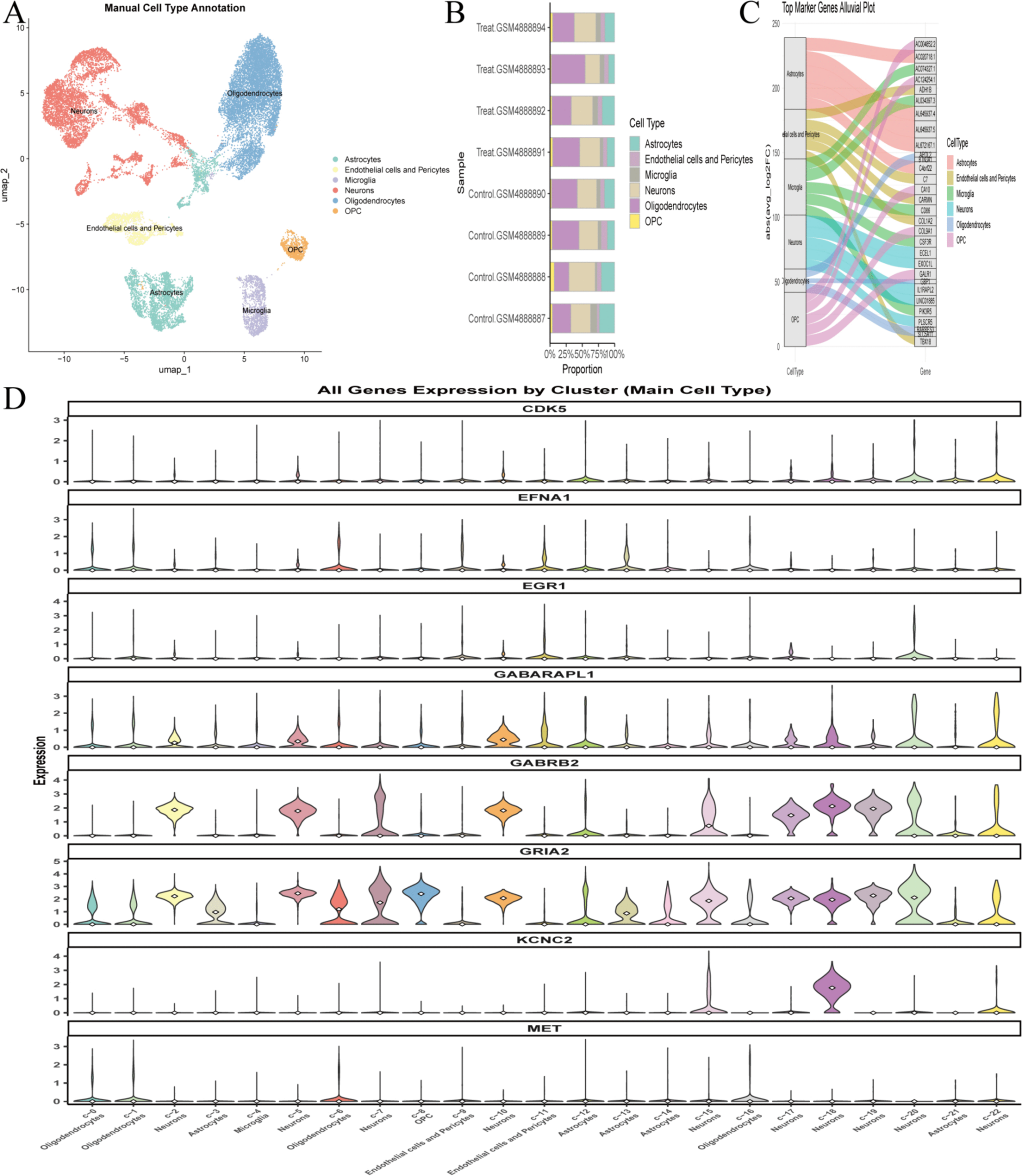
Cell Type Annotation and Core Gene Expression Characteristic Analysis of scRNA-seq Data. **(A)**. UMAP plot of annotated cell types (Astrocytes, Endothelial cells/Pericytes, Microglia, Neurons, Oligodendrocytes, OPC), clarifying the main cell populations involved in AD pathology. **(B)**. Bar chart of cell type proportion in each sample, showing cell composition changes in AD brain. **(C)**. Sankey diagram of the top 10 marker genes per cell type, displaying cell-specific marker genes. **(D)**. Violin plot of core gene expression across cell clusters, revealing the cell-type-specific expression profile of core genes, and providing a cellular basis for TSA’s regulatory mechanism in AD.

Single-cell sequencing data were subjected to strict quality control (Fig 8A,B). After normalization and identification of 2500 highly variable genes (Fig 8C), PCA and UMAP dimensionality reduction (15 principal components, resolution = 0.6) identified 22 cell clusters (Fig 8D,E), annotated as Astrocytes, Endothelial cells/Pericytes, Microglia, Neurons, Oligodendrocytes, and OPC (Fig 9A). A bar chart showed the proportion of various cell types in different samples (Fig 9B), and a Sankey diagram showed the top 10 marker genes of each cell type and their quantities (Fig 9C). Core genes showed cell-type-specific expression: MET was highly expressed in Oligodendrocytes; CDK5, KCNC2, GRIA2, and GABRB2 were enriched in Neurons; EFNA1 was expressed in Endothelial cells, Pericytes, Oligodendrocytes, and Astrocytes; EGR1 and GABARAPL1 were detected in Endothelial cells, Pericytes, and Neurons (Fig 9D). These results provide important information for us to understand the transcriptomic characteristics of different cell types in Alzheimer’s disease, revealing the expression profiles of characteristic genes in various cell populations and their potential roles in the disease. These findings will lay the foundation for the development of more precise therapeutic strategies in the future, focusing on effective intervention for functional changes in specific cell types (S7 Table).

### 3.8. Molecular docking and molecular dynamics simulation

**Fig 10:**
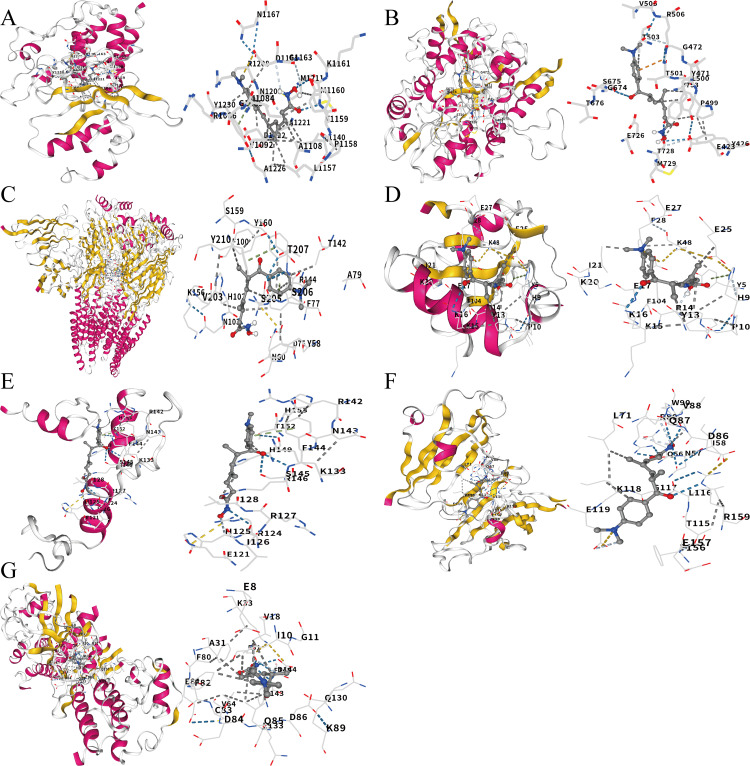
Molecular docking analysis of the binding between TSA and 7 core AD target proteins performed via CB-Dock2 (AutoDock Vina v1.1.2). **(A-G).** Molecular docking conformation diagrams of complexes formed by TSA binding to MET, GRIA2, GABRB2, GABARAPL1, EGR1, EFNA1 and CDK5 proteins, respectively. Receptor: ribbon model; TSA: stick model; yellow dashed lines: hydrogen bonds.

**Fig 11:**
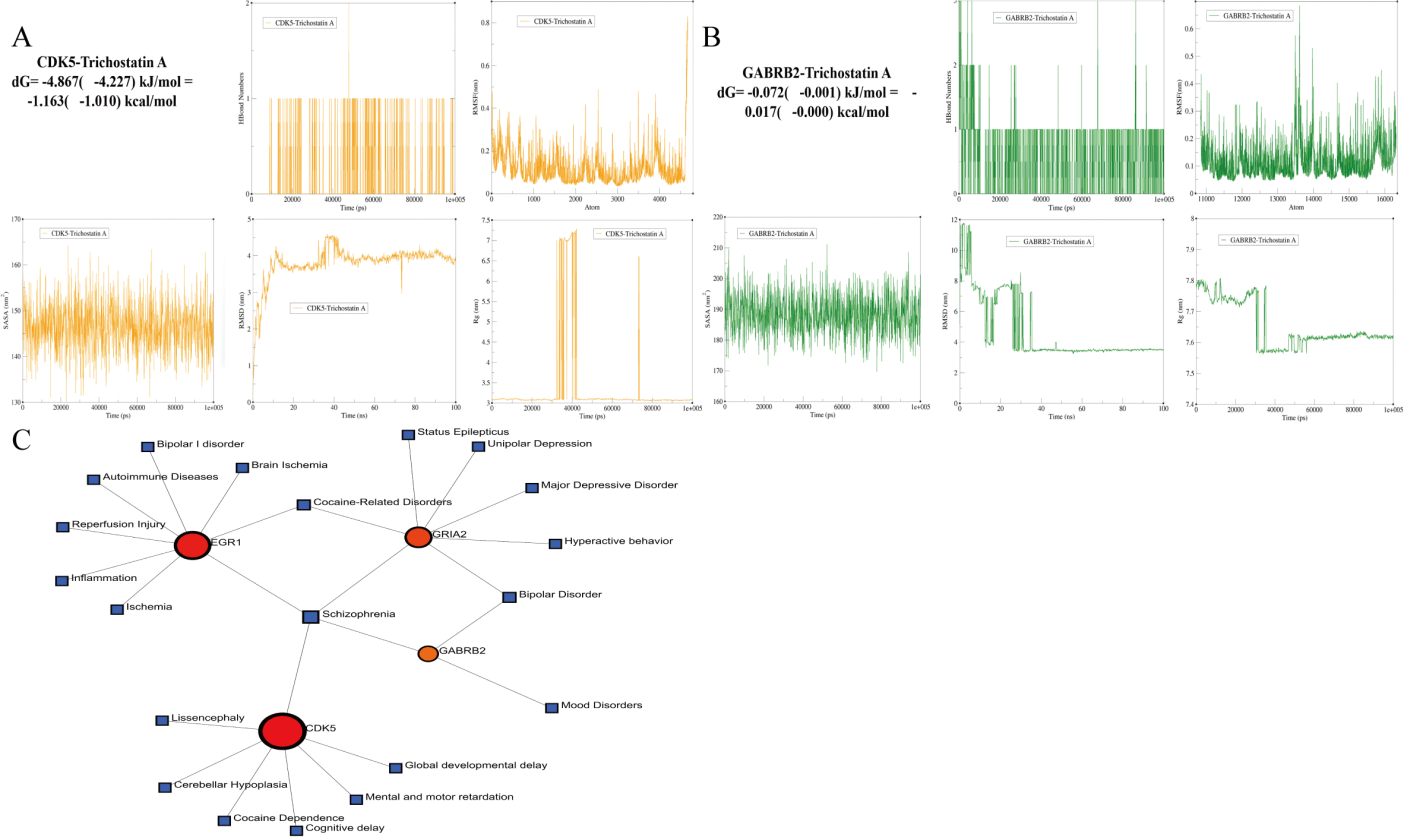
100 ns MD Simulation Analysis and Disease Prediction Based on Core Genes. **A**. Dynamic parameters of TSA-CDK5 complex during 100 ns MD simulation: protein backbone RMSD stabilized within 0.3 nm after 40 ns; hydrogen bond count averaged 1 (0–2 fluctuations); Rg maintained at ~3.42 nm; SASA fluctuated around 147 nm²; most residue RMSF < 0.3 nm; MMPBSA binding energy dG = −1.163 kcal/mol, confirming weak binding affinity. **B**. Dynamic parameters of TSA-GABRB2 complex during 100 ns MD simulation: protein backbone RMSD stabilized at ~0.25 nm after 40 ns; hydrogen bond count averaged 1 (0–3 fluctuations); Rg maintained at ~7.63 nm; SASA fluctuated around 190 nm²; most residue RMSF < 0.3 nm; MMPBSA binding energy dG = −0.017 kcal/mol, confirming weak binding affinity. **C**. Disease prediction result plot based on core genes and the DisGeNET database, verifying the close association between core genes and AD-related neurodegenerative diseases.

Molecular docking was performed on 7 core genes (excluding KCNC2, no high-resolution 3D structure) with TSA. All 7 proteins had binding energies < −5 kcal/mol (Table 2), indicating predicted stable interactions (Fig 10A-G). TSA exhibited the highest binding affinity with GABRB2 (−9.7 kcal/mol) and CDK5 (−8.9 kcal/mol), which were selected for MD simulation. MD results revealed weak but stable binding: TSA-CDK5 complex: RMSD stabilized at ~0.3 nm after 40 ns, average 1 hydrogen bond, binding energy dG = −1.163 kcal/mol (Fig 11A); TSA-GABRB2 complex: RMSD stabilized at ~0.25 nm post-40 ns, average 1 hydrogen bond, binding energy dG = −0.017 kcal/mol (Fig 11B). These findings suggest weak direct binding between TSA and core proteins under physiological conditions, supporting that TSA may regulate core genes primarily through epigenetic mechanisms (consistent with its HDAC inhibitor property).

**Table 2:** Binding energies of ligands and receptors.

Ligand	Receptor	Bind. energy [kcal/mol]
TSA	GABRB2	−9.7
TSA	CDK5	−8.9
TSA	MET	−8.6
TSA	GRIA2	−8.0
TSA	EFNA1	−7.7
TSA	GABARAPL1	−6.7
TSA	EGR1	−5.8

### 3.9. Disease and drug prediction

DisGeNET database analysis showed 8 core genes were significantly associated with AD-related diseases (schizophrenia, bipolar disorder, status epilepticus, etc.), involving neurotransmitter homeostasis and immune-inflammatory responses (Fig 11C). DSigDB prediction identified key candidate drugs: roscovitine (CDK5 inhibitor) and oxazepam (GABRB2 modulator) (most significantly associated with core genes), consistent with TSA’s mechanism in regulating tau pathology and GABAergic transmission (Table 3). Molecular docking identified the binding characteristics of 24 pairs involving 14 ligands and 7 core AD receptors, and 3 high-affinity binding pairs were screened out: roscovitine-CDK5 (−11.6 kcal/mol), Staurosporine-MET (−9.9 kcal/mol), and (-)-Epigallocatechin gallate-MET (−9.2 kcal/mol). This provides crucial data support for the screening of AD-targeted drug candidates and mechanism validation (S8 Table)(S9 Table).

**Table 3:** Predicted drug candidates table.

Term	Adjusted P-value	Combined Score	Genes
trichostatin A	0.04357029387737973	38.637728138647375	EFNA1;GABRB2;EGR1;GRIA2;MET
roscovitine	0.04357029387737973	474.9953903533844	CDK5
oxazepam	0.04357029387737973	725.5830081396662	GABRB2
valproic acid	0.04357029387737973	44.524546399274314	EFNA1;GABRB2;EGR1;GRIA2;CDK5;GABARAPL1;MET
topiramate	0.009345834166944948	1266.5151113505071	GABRB2;GRIA2
felbamate	0.04357029387737973	1138.8637280375192	GABRB2
Crizotinib	0.04357029387737973	643.5130349149837	MET
parthenolide	0.00603526786226265	513.2655280213447	EFNA1;EGR1;GABARAPL1
Staurosporine	0.04357029387737973	105.53099370120802	CDK5;MET
LY-294002	0.04357029387737973	643.5130349149837	EGR1
okadaic acid	0.04566238170901712	260.68981695680077	CDK5
4-aminobutyric acid	0.04357029387737973	643.5130349149837	GABRB2
quercetin	0.044802965299605126	371.8308142207318	EGR1
(-)-Epigallocatechin gallate	0.04357029387737973	435.442406227662	MET
Acamprosate calcium	0.04357029387737973	576.7477724354015	GABRB2

## 4. Discussion

This study systematically investigated the potential molecular network of TSA in regulating AD and identified 8 candidate core regulatory genes, which provided preliminary theoretical clues for the potential therapeutic value of TSA in AD. Meanwhile, it suggested that multi-level associations may exist among multiple links such as immune microenvironment imbalance and transcriptional regulation disorder during AD pathogenesis, offering potential novel molecular biomarkers and candidate intervention targets for the early diagnosis and targeted therapy of AD.

TSA has 949 potential targets involved in metabolism, signal transduction and neural function, in line with its broad-spectrum HDAC inhibitor property—HDACs regulate gene transcription, cell cycle and neuroprotection via histone and non-histone acetylation [22]. Core genes were enriched in key AD-related pathways (GABAergic synapse, neuroactive ligand-receptor interaction, MAPK), which are closely linked to AD pathogenesis [23,24]. Aberrant MAPK activation induces neuronal apoptosis in AD [25], and TSA’s target genes in this pathway may alleviate neuronal damage by inhibiting excessive activation, consistent with HDAC inhibitors’ neuroprotective effects [26]. Core genes GABRB2, GABARAPL1, GRIA2 and MET, which are markedly dysregulated in AD [27,28], participate in GABAergic and neuroactive ligand-receptor pathways, implying TSA may epigenetically regulate their expression to restore neurotransmitter balance [29,30]. For tau pathology, CDK5 (a key tau phosphorylation kinase) had high binding affinity with TSA in molecular docking, but MD simulations confirmed weak direct binding, supporting TSA inhibits abnormal CDK5 activation via epigenetic mechanisms [31].

The eight candidate core genes display an antagonistic imbalance pattern in AD—synergistic downregulation of protective genes and sole upregulation of the pathological gene EFNA1—and are implicated in core AD pathological processes including tauopathy, neurotransmission disorder, synaptic damage and neuroinflammation. TSA may regulate these genes not via direct binding but through epigenetic targeting of HDAC1–3 and HDAC6, repairing gene interaction imbalance to intervene in AD progression. For tau pathology and autophagic clearance, the imbalance between abnormal CDK5 activation and GABARAPL1 downregulation mediates abnormal tau phosphorylation and autophagic dysfunction [32–34]. TSA may indirectly inhibit aberrant CDK5 kinase activity by suppressing HDAC1 and regulating its acetylation, while targeting cytoplasmic HDAC6 to enhance GABARAPL1-mediated autophagic regulation, promoting abnormal tau degradation and forming a potential “TSA-HDAC1/6-CDK5/GABARAPL1” regulatory axis for tauopathy intervention. At the neurotransmission level, GABRB2 and KCNC2 are co-expressed in GABAergic interneurons to maintain neuronal excitation-inhibition balance; their synchronous downregulation impairs GABAergic inhibitory transmission and exacerbates electrophysiological disorders [35–37]. TSA may target class I HDACs (HDAC1–3) to upregulate GABRB2 expression, thereby restoring inhibitory postsynaptic current function [38,39], and presumably regulate KCNC2 via the same HDAC subtypes to restore brain excitation-inhibition homeostasis, despite no direct experimental evidence for HDAC-KCNC2 interaction. For synaptic plasticity and neurotrophy, synergistic downregulation of EGR1 (synaptic plasticity-related transcription) and MET (myelin integrity) exacerbates cognitive impairment, and both may be antagonized by EFNA1 [40–42]. TSA may epigenetically upregulate their expression to antagonize the neurotoxic effect of EFNA1, thereby restoring synaptic plasticity and myelin homeostasis [43,44]. In addition, GRIA2 and EFNA1 jointly regulate synaptic integrity: downregulation of GRIA2 can induce α-amino-3-hydroxy-5-methyl-4-isoxazolepropionic (AMPA) receptor calcium overload and synaptic loss [45,46], while upregulation of EFNA1 interferes with axon guidance and exacerbates synaptic damage [47]. It is speculated that TSA can epigenetically regulate the expression of both (downregulating EFNA1 and upregulating GRIA2) to improve synaptic destruction [48]. As the only upregulated pathological gene, EFNA1 can antagonize the function of other protective genes through multiple mechanisms, forming a vicious cycle of amplified neuroinflammation [49]. TSA can target HDAC6 to enhance autophagic degradation efficiency, promoting the clearance of abnormal proteins such as EFNA1; meanwhile, it synergizes with GABARAPL1 to strengthen the “autophagy-inflammation” balance repair, blocking the vicious cycle between neuroinflammation and neurodegeneration [50–52]. In summary, TSA precisely targets specific subtypes including HDAC1–3 and HDAC6 to regulate the synergistic-antagonistic network of core genes in the four major pathological modules of AD, establishing a multi-dimensional epigenetic intervention system for AD pathological repair. This study initially clarifies the potential molecular pathway of TSA in regulating AD, providing directions for subsequent verification of HDAC subtype-specific effects and design of combination intervention strategies.

MD simulation results showed that TSA-CDK5 and TSA-GABRB2 complexes stabilized in the late simulation stage, but no stable direct binding was formed between TSA and these two proteins, which was inconsistent with the high-affinity molecular docking prediction. This indicates that TSA likely regulates CDK5 and GABRB2 via its core HDAC inhibitor function—epigenetic regulation such as relieving histone deacetylation-mediated gene promoter transcriptional inhibition—thereby upregulating GABRB2, inhibiting abnormal CDK5 activation, improving GABAergic neurotransmission and reducing tauopathy [53,54]. This mechanism may explain the tauopathy intervention effect of low-dose TSA, and TSA’s synergistic upregulation of GABARAPL1 and GABRB2 may further enhance inhibitory synaptic function [55]. In conclusion, MD simulation results suggest that TSA’s regulation of CDK5 and GABRB2 may mainly rely on epigenetic mechanisms, providing structural biological clues for understanding its potential multi-target intervention mechanism in AD.

The potential synergistic regulatory network formed by the 8 candidate core genes and their functionally associated diseases may highlight the evolutionary conservation of AD pathological mechanisms: CDK5 is associated with neurodevelopment-related diseases, confirming its core role in neurodevelopment and cognitive formation [56]. Bipolar disorder and other mood disorders are associated with GABRB2 and GRIA2, highlighting the conserved role of the GABAergic and glutamatergic systems in emotion and cognitive regulation [57,58]. These associations can provide references for understanding the conservation of core AD pathological mechanisms. In addition, the drugs identified in this study corroborate the functions of candidate core genes and the potential regulatory mechanisms of TSA from multiple dimensions: roscovitine synergistically inhibits tau pathology with TSA by targeting CDK5, which may form an additive effect with TSA’s epigenetic inhibition of CDK5 [59]; oxazepam enhances inhibitory transmission with GABA through GABRB2 [60], which can supplement TSA’s epigenetic regulation mechanism of upregulating GABRB2 expression; valproic acid and topiramate regulate synaptic homeostasis [61,62], and neuroprotective anti-inflammatory drugs such as parthenolide exert their respective effects [63], providing references for AD multi-target therapy exploration.

The AD immune microenvironment is characterized by abnormal infiltration of plasma cells and CD8 + T cells, and the upregulated EFNA1 is positively correlated with these immune cells, suggesting it acts as a mediator of immune-neural crosstalk in AD neuroinflammation [64]. Downregulation of other core genes (GABRB2, GABARAPL1, EGR1) and decreased MET expression may indirectly exacerbate neuroinflammation and disrupt immune homeostasis [65–67], and TSA’s epigenetic regulation of these genes may synergistically improve AD immune imbalance. Combined with previous evidence that EFNA1 regulates immune cell migration and activation via Eph receptors [68], TSA may epigenetically inhibit abnormal EFNA1 upregulation and upregulate other core genes to reduce excessive immune activation in AD, forming a “TSA-core genes-immune cells” regulatory axis that provides a new perspective for understanding AD neuroinflammation-neurodegeneration crosstalk.

The ceRNA network and transcription factor analysis (with ZNF805 and ZNF385A as major regulatory factors) constructed in this study revealed the multi-level regulatory patterns of the core genes. For example, the expression of GABRB2 may be epigenetically regulated by the lncRNA-miRNA axis [69]. As an HDAC inhibitor, TSA may synergistically upregulate core genes such as GABRB2 by relieving histone deacetylation-mediated transcriptional inhibition, consistent with the epigenetic-dominated mechanism confirmed by MD simulations. This epigenetic-transcriptional-post-transcriptional synergistic regulation reflects TSA’s overall intervention potential on the AD molecular network. The eight core genes exhibit distinct cell-type-specific expression in the AD brain: MET is highly expressed in oligodendrocytes (related to myelin formation) [70]; CDK5 and GRIA2 are enriched in neurons (related to synaptic function) [71,72]; EFNA1 is expressed in endothelial cells, pericytes, oligodendrocytes, and astrocytes (may be involved in blood-brain barrier function regulation and glial cell crosstalk) [73]; EGR1 and GABARAPL1 are expressed in endothelial cells, pericytes, and neurons (suggesting their potential role in neurovascular unit function regulation) [74]; KCNC2 is mainly localized in neurons (closely related to neuronal electrophysiological activity) [75]. These cell type-specific expression characteristics suggest that TSA may target candidate core genes in different cell types (coordinating intercellular functions through epigenetic regulation), providing the possibility of simultaneously improving multiple pathological changes in AD and offering cellular-level clues for understanding TSA’s potential multi-target intervention mechanism.

This study explicitly acknowledges that the functions of candidate core genes, the epigenetic mechanism of TSA in regulating AD, and the immune-neural crosstalk mode all lack experimental evidence from cell and animal models; additionally, HDAC subtype-specific effects of TSA and tissue-level verification of immune infiltration remain uncompleted, which may affect the reliability and clinical translation of the conclusions. Despite these limitations, this study innovatively identified the TSA-regulated AD core gene network, epigenetic-dominated multi-target intervention mode, and core gene cell-type-specific expression via integrated bioinformatics and machine learning, providing a novel perspective for AD mechanism research and targeted therapy. All in silico findings require experimental validation to confirm their biological significance, and future research will focus on: 1) CRISPR-mediated core gene modulation in AD cell models to detect synaptic function, p-Tau levels and neuronal survival; 2) TSA intervention in 5 × FAD and other AD transgenic mice to verify the epigenetic regulatory chain, including TSA’s inhibition of HDAC1/3 and the specific acetylation sites of core gene promoters; 3) immune cell-neuron co-culture and brain tissue immunofluorescence to confirm EFNA1-mediated immune-neural crosstalk and core gene localization correlation. Systematic experimental verification will further consolidate the scientificity of the findings and provide a more reliable experimental basis for AD targeted therapy.

## 5. Conclusion

This study explored the putative intervention mechanism of the HDAC inhibitor trichostatin A (TSA) in AD via integrated bioinformatics, machine learning, molecular docking and MD simulations. We identified 949 potential TSA targets overlapping with AD-related differential genes, and screened eight core genes with distinct AD expression patterns and involvement in key AD pathological processes via machine learning. MD simulations imply TSA may not stably bind these candidates; its regulation might tentatively rely on epigenetic mechanisms targeting HDAC1–3/6, potentially restoring gene network balance and interrupting neuroinflammation-neurodegeneration cycles. Additional analyses revealed the complex regulation and cell-type-specific expression of these core genes. This work provides initial clues for TSA’s AD intervention potential, clarifies the tentative diagnostic/therapeutic value of the eight core genes, and deepens understanding of the AD molecular network. Notably, all in silico findings require further experimental verification for clinical translation.

## Supporting information

S1 Table Predicted Targets of TSA from ChEMBL, SwissTargetPrediction and PharmMapper. (XLSX)

S1 Fig Gene expression correlation matrix of 8 target genes (EFNA1, GABRB2, GABARAPL1, EGR1, CDK5, KCNC2, MET, and GRIA2). (DOCX)

S2 Table DEG Analysis Results of AD Datasets and Gene Lists of Each Module from WGCNA. (XLSX)

S2 Fig Ligand-receptor binding combinations corresponding to each subfigure. (DOCX)

S3 Table KEGG, DO and GO Enrichment Analysis Results of Intersection Genes. (XLSX)

S4 Table ROC Values of Models Constructed by 130 Machine Learning Algorithms and Genes Selected by Each Model. (XLSX)

S5 Table AD Immune Infiltration Results, Immune Infiltration Differential Analysis Results, and Correlation Analysis Between Model Genes and Immune Cells. (XLSX)

S6 Table GSEA Results of Model Genes. (XLSX)

S7 Table Marker Genes for Single-Cell Annotation, Cell Cluster Annotation Results, and Expression Results of Model Genes Across Different Cell Types. (XLSX)

S8 Table Predicted Potential Associated Drugs for Model Genes Based on the DSigDB Database. (XLSX)

S9 Table Molecular Docking Results of Drugs Selected from Table 3 and Model Genes. (XLSX)
