# Supplementary material for: Deciphering the molecular network of Trichostatin A in regulating Alzheimer’s disease screening of core genes and mechanistic investigation based on multidimensional bioinformatics and molecular simulation
Source: PLoS One. 2026 Apr 20;21(4):e0347532. doi: 10.1371/journal.pone.0347532 (PMC13094961; doi:10.1371/journal.pone.0347532)
Supplement: S1 Fig — (DOCX) [file pone.0347532.s002.docx]

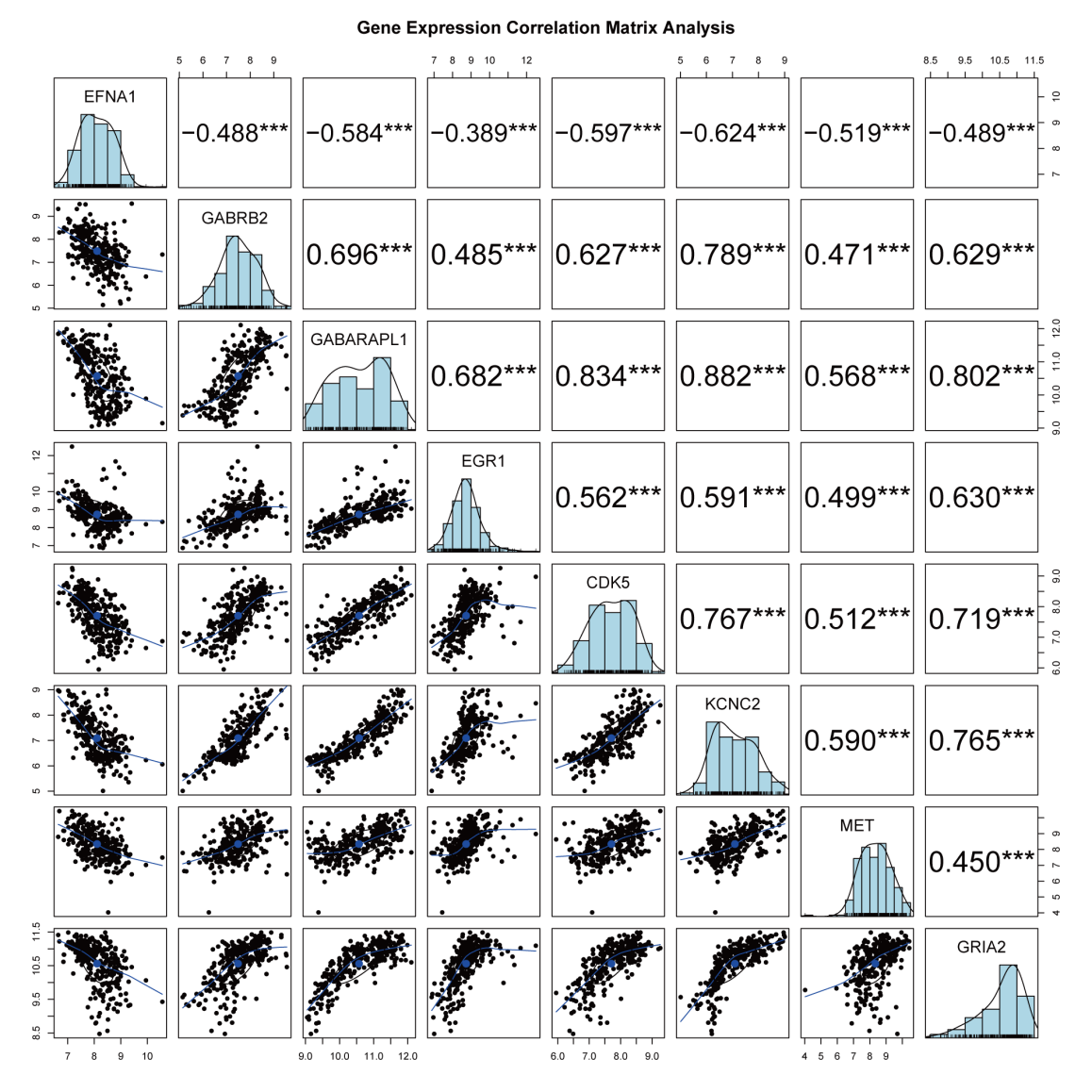


**S1 Fig. Gene expression correlation matrix of 8 target genes (EFNA1, GABRB2, GABARAPL1, EGR1, CDK5, KCNC2, MET, and GRIA2).** The values in the matrix represent the correlation coefficients between gene pairs, with *** indicating extremely significant correlation (*P* < 0.001). The correlation coefficients range from -0.624 to 0.882, reflecting the strength and direction of the association in expression levels among the genes.
