# Supplementary material for: Deciphering the molecular network of Trichostatin A in regulating Alzheimer’s disease screening of core genes and mechanistic investigation based on multidimensional bioinformatics and molecular simulation
Source: PLoS One. 2026 Apr 20;21(4):e0347532. doi: 10.1371/journal.pone.0347532 (PMC13094961; doi:10.1371/journal.pone.0347532)
Supplement: S2 Fig — (DOCX) [file pone.0347532.s004.docx]

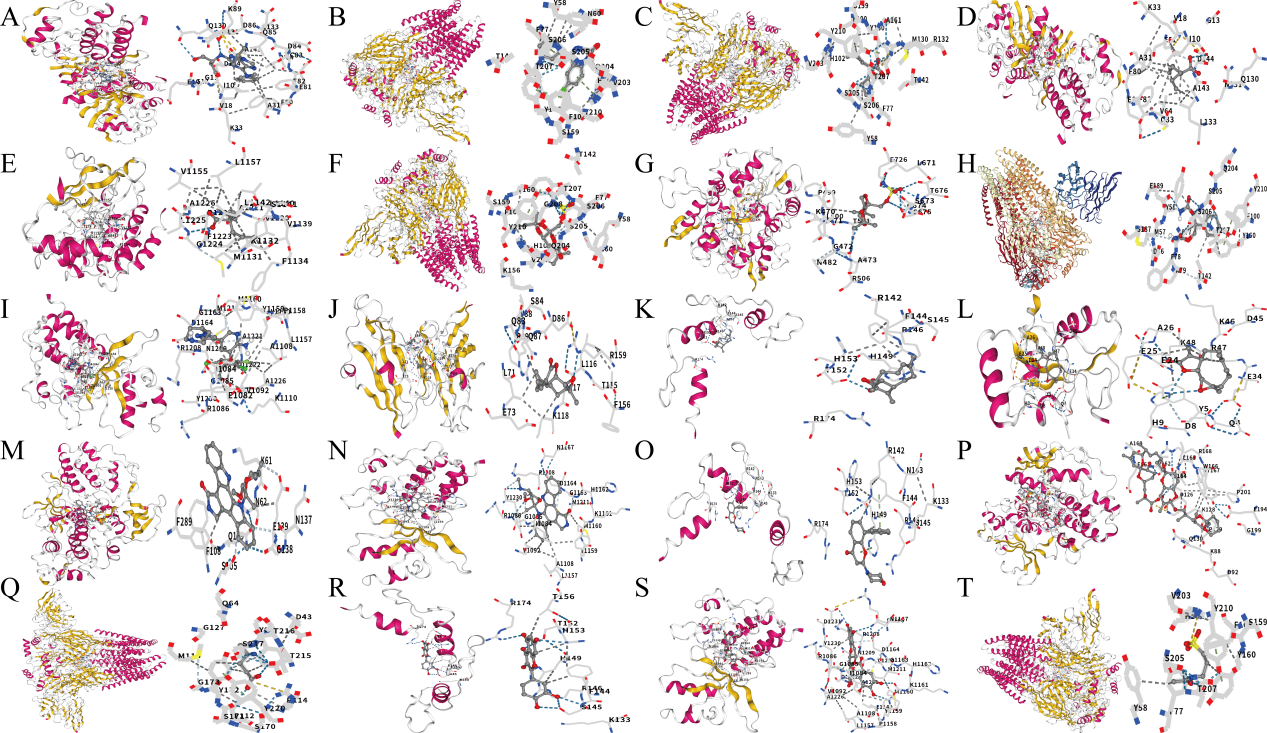


**S2 Fig. Ligand-receptor binding combinations corresponding to each subfigure.** **A**. ligand roscovitine and receptor CDK5; **B**. ligand oxazepam and receptor GABRB2; **C**. ligand valproic acid and receptor GABRB2; **D**. ligand valproic acid and receptor CDK5; **E**. ligand valproic acid and receptor MET; **F**. ligand topiramate and receptor GABRB2; **G**. ligand topiramate and receptor GRIA2; **H**. ligand felbamate and receptor GABRB2; **I**. ligand Crizotinib and receptor MET; **J**. ligand parthenolide and receptor EFNA1; **K**. ligand parthenolide and receptor EGR1; **L**. ligand parthenolide and receptor GABARAPL1; **M**. ligand Staurosporine and receptor CDK5; **N**. ligand Staurosporine and receptor MET; **O**. ligand LY-294002 and receptor EGR1; **P**. ligand okadaic acid and receptor CDK5; **Q**. ligand 4-aminobutyric acid and receptor GABRB2; **R**. ligand quercetin and receptor EGR1; **S**. ligand (-)-Epigallocatechin gallate and receptor MET; **T**. ligand Acamprosate calcium and receptor GABRB2.
